# 

**Published:** 1874-07-15

**Authors:** 


### NEW BOOKS RECEIVED.

*From Jansen, McClurg & Co.*

MATERIA MEDICA FOR THE USE OF STUDENTS. By John B. Biddle, M.D. Sixth edition, revised and enlarged. Lindsey & Blakeston, Philadelphia. Price, cloth, $4.00.

---

A CONSPECTUS OF MEDICAL SCIENCES, comprising Manuals of Anatomy, Physiology, Chemistry, Materia Medica, Practice of Medicine, Surgery and Obstetrics, for the use of Students. By Henry Hartshorne, A.M., M.D. Second edition, enlarged and thoroughly revised. Henry C. Lea, Philadelphia.

With both of the above works the profession is thoroughly familiar through their former editions. For their present issue, they have been throughly revised and some considerable matter added to each, bringing them up to date in the new advancements and discoveries.

---

INFLAMMATION OF THE LUNGS, TUBERCULOSIS AND CONSUMPTION. Twelve Lectures by Dr Ludwig Buhl. Translated from the second German edition. New York: G. P. Putnam's Sons, 1874. Price, $1.75.

